# Outcomes of hysterectomy for puerperal sepsis at an Academic Hospital: A retrospective study

**DOI:** 10.4102/jcmsa.v3i1.216

**Published:** 2025-08-30

**Authors:** Esther Olusola, Admire Chikandiwa, Evelyn Ngwa Lumngwena, Poovangela Naidoo

**Affiliations:** 1 Department of Obstetrics and Gynaecology, Faculty of Health Sciences, University of the Witwatersrand, Johannesburg, South Africa; 2 School of Clinical Medicine, Faculty of Health Sciences, University of the Witwatersrand, Johannesburg, South Africa; 3 Centre for the Study of Emerging and Re-emerging Pathogens, Institute of Medical Research and Medicinal Plant Studies, Ministry of Scientific Research and Innovation, Yaounde, Cameroon

**Keywords:** postpartum, puerperal sepsis, hysterectomy, bacterial strains, antibiotic resistance

## Abstract

**Background:**

Pregnancy-related sepsis contributes significantly to maternal mortality. While there is substantial information on postpartum hysterectomy, information on outcomes of hysterectomy as source control for puerperal sepsis is limited. Knowledge of the common causative organisms and their antimicrobial sensitivity may assist with targeted antibiotic therapy to improve patient outcomes. This study described the indications and outcomes of surgery in patients following hysterectomy as source control for puerperal sepsis.

**Methods:**

In a retrospective study, we analysed the intra-operative and histological findings and the results of microbial culture and antibiotic sensitivity of women who underwent a hysterectomy for puerperal sepsis at Chris Hani Baragwanath Academic Hospital (CHBAH), Johannesburg, from January 2019 to December 2019.

**Results:**

Twenty-nine (88%) of the 33 women with hysterectomy for puerperal sepsis studied had a caesarean section (CS), 14 (48%) of whom had a CS performed for foetal distress. Eight of these 33 women (24%) had hypertensive-related disorders. The most common organisms cultured in the intra-abdominal fluid were *Acinetobacter baumannii* (*n* = 11, 26%), *E. coli* (*n* = 8, 19%), *Klebsiella* species (*n* = 6, 14%) and *Enterococcus faecalis* (*n* = 6, 14%) of a total of 42 organisms were identified from all sites. Puerperal sepsis was confirmed in 28 (85%) of the uterine histology samples, with a mortality rate of 6% in this study.

**Conclusion:**

Hysterectomy for puerperal sepsis was most frequently associated with CS, with hypertensive-related disorders the most common indication. Histological confirmation of sepsis is required, as the histological findings differed by 15% with surgical diagnosis.

**Contribution:**

*A. baumannii* was the most common species isolated as the cause of infection.

## Introduction

Sepsis during pregnancy and specifically in the puerperal phase is one of the leading causes of maternal morbidity and mortality worldwide, accounting for up to 11% of maternal deaths in developing countries.^1,2,3,4,5^ In the 2023 South African Saving Mothers Report (SASMR), 4.9% maternal deaths were because of pregnancy-related sepsis (PRS), which is similar to our institutional mortality rate of 5%.^6^ This is similar to the 5% mortality rate of severe sepsis in developed countries.^7^ A further 17.1% maternal deaths because of non-pregnancy related infection were reported from the SASMR.^6^ The total number of maternal deaths attributed to sepsis has steadily decreased from 70 in 2017 to 44 in 2023.^6,8^ The SASMR of 2017 outlined PRS as ‘often underestimated by the healthcare provider’ with inadequate management.^8^ Although there has been a steady decline, there are still many avoidable deaths because of PRS. Puerperal sepsis, as defined by the World Health Organization, is any infection occurring from the onset of labour until 42 days postpartum.^9^ Clinically, it is defined by the presence of a fever and one of the following: abnormal vaginal discharge, pelvic pain, offensive discharge or a delay in involution of the uterus.^6,9,10^ A favourable outcome is dependent on early recognition, timeous and appropriate commencement of antibiotic therapy and source control.^11^ However, hysterectomy is also indicated when sepsis is complicated by the presence of disseminated intravascular coagulopathy, or when there is dysfunction of two or more end organs.^8^ The fifth SASMR found that delayed diagnosis and a low proportion of hysterectomies (14%) were suggestive of a delay in definitive treatment of the women who died from PRS.^12^ This SASMR found the final cause of death in 90% of PRS deaths to be septic shock.^12^ The implementation of the recommendations of this report, which encouraged early recognition and timeous hysterectomy, has seen a steady decline in maternal mortality as a result of PRS.^12^

A study conducted between 2007 and 2009 in the Eastern Cape revealed that secondary postpartum haemorrhage and puerperal sepsis accounted for 27% of the peripartum hysterectomies.^13^ In 2025, Mmabatswa et al. found that 32.1% of postpartum hysterectomies resulted from PRS in a study of 56 women who underwent peripartum hysterectomy at the Johannesburg General Hospital between 2018 and 2020. Almost 88.8% of these PRS were histopathologically confirmed as sepsis.^14^

Several risk factors have been identified as contributors to maternal sepsis. The predominant ones include the human immunodeficiency virus (HIV) seropositivity, prolonged rupture of membranes, prolonged labour, prolonged surgery, the presence of chorioamnionitis, previous surgery and bowel injury.^12^

Obstetric-related infections in the antepartum period include septic abortions and chorioamnionitis, while endometritis and wound infections occur in the postpartum period.^15^ Non-obstetric sources of infection in the antepartum and postpartum periods have predominantly been described in the urinary and respiratory tract.^9,16^ The Global Maternal Sepsis Study found urinary tract, genital tract (endometritis), chorioamnionitis and respiratory tract infections as the most common maternal infections.^1,16^ Other potential risk factors for puerperal sepsis include caesarean sections (CS), postpartum haemorrhage, preterm deliveries and comorbid conditions, such as diabetes and eclampsia.^3^

According to the fifth SASMR, avoidable factors, such as an overburdened healthcare system with a lack of facilities, led to missed opportunities for preventing maternal deaths from PRS. Thirty-three per cent of PRS deaths developed puerperal sepsis after a CS.^12^ In 2010, a longitudinal descriptive study of puerperal infection found no risk identifying factors, which is contradictory to the findings of Mmabatswa et al.^14,17^

While maternal mortality because of puerperal sepsis has been reduced by antibiotics coverage in developed countries, there is limited information on the common bacterial strains involved in South Africa and the potential sensitivity patterns amid the growing incidence of antibiotic resistance. Evidence of common infectious patterns and antibiotic sensitivity patterns is required for appropriate patient management and for antibiotic stewardship.^2^ This study aimed to describe the demographic, intra-operative findings, organisms responsible for infection, the antimicrobial susceptibility patterns and the histological findings of women who had a hysterectomy as a measure of source control in the treatment of PRS.

## Research methods and design

This was a retrospective cross-sectional review of medical records at the Obstetrics unit of the Chris Hani Baragwanath Academic Hospital (CHBAH) situated in Soweto, Johannesburg, between 01 January and 31 December 2019. This is an Obstetrics unit of a tertiary teaching hospital, where women from peripheral clinics are referred. The study involved all women who underwent a hysterectomy for puerperal sepsis and whose samples were sent for histology and suspected endometritis during this study period. Women who had a miscarriage or a pregnancy at ≤ 24 weeks’ gestation were excluded. Demographic and clinical data, including comorbidities, previous surgical history, duration of labour, mode of delivery, surgical, microbiology data and sites, dissemination of intravascular coagulation from post-mortem findings and histological reports were extracted and analysed. The duration of CS was identified through theatre notes and categorised accordingly. Frequencies and percentages were used to describe the findings, including caesarean and hysterectomy characteristics, among others. Age, parity, gravity and gestational ages were summarised with medians and interquartile ranges (IQR). Differences between proportions were assessed using Fisher’s exact test. The differences between admission, pre-hysterectomy and post-hysterectomy values were evaluated using ANOVA (analysis of variance) for repeated measures test with a *p*-value of < 0.05 considered statistically significant using STATA software (StataCorp version 15).

### Ethical considerations

Ethical clearance (No. M200979) was obtained from the University of the Witwatersrand Human Research Ethics Committee (Medical) on 4 December 2020, and all permissions were obtained from the hospital administration before the commencement of the study. Because of the retrospective nature of the study, individual participant consent was not possible, but pseudanonymisation was used to protect participant confidentiality during analyses and publication.

## Results

During the same period, 33 hysterectomies were performed for puerperal sepsis (Table 1), representing 0.17% (*n* = 33/18 458) of all deliveries at this study site. Forty-five per cent of all deliveries were CS. The median gestational age of the study population at the time of delivery was 38 weeks (range 34 to 40), with a median body mass index (BMI) of 28 kg/m^2^ (range 34 to 40). Seven (21%) women were HIV seropositive and eight women (24%) had hypertensive-related disorders (Table 1).

**TABLE 1:** Demographic characteristics of the study population (*N* = 33).

Variable	Median (IQR)	%
Age in years	28	22–34
Parity	1	0–2
Gravidity	2	1–4
Gestational age at delivery in weeks	38	34–40
**Race**		
Black people	27	82
Mixed race people	6	18
BMI (kg/m^2^)	28	26–31
RPR	2	6
HIV seropositive	7	21
**Comorbidities**		
Hypertensive	5	15
Pre-eclampsia	3	9
Cardiac	2	6
Diabetes	1	3

BMI, body mass index; RPR, rapid plasma reagin; HIV, human immunodeficiency virus.

### Mode of delivery characteristics

Of the 33 women who had puerperal sepsis cases, 29 (88%) had a CS and four (12%) had a normal vaginal delivery. The most prevalent indication for CS in these women was foetal distress (*n* = 14, 48%). The description of ‘other’, which accounted for 31% of the women in the study (Table 2), included 4 (14%) women with antepartum haemorrhage (uterine rupture and placenta previa), 1 (3.5%) eclampsia, 1 (3.5%) cephalopelvic disproportion and 3 (10%) elective CS.

**TABLE 2:** Indications for the mode of delivery characteristics and duration of Caesarean Section (*N* = 29).

Indications for caesarean section	*n*	%
**Indication**		
Foetal distress	14	48
Poor progress	2	7
Previous caesarean section in labour	4	14
Other	9	31
**Operative times (min)**		
0–30	4	14
30–60	15	52
> 60	4	14
Unknown	6	20

### Surgical characteristics of women who had puerperal sepsis

The duration of CS ranged between 30 min and 60 min in 50% of women, with half either less than 30 min or greater than 60 min, respectively (Table 2). Twenty-nine women (88%) had a total abdominal hysterectomy (TAH) in comparison to 4 (12%) who had a subtotal hysterectomy (STAH) (Table 3). Two of those with STAH recovered uneventfully and were discharged home. One had ongoing sepsis and required cervical stump removal and eventually recovered. The fourth woman who had an STAH demised because of ongoing sepsis with *Acinetobacter baumannii* bacteraemia. Post-mortem results on this woman further revealed she had a necrotic cervix. One of the women post-TAH demised because of deep incision site infection, and the post-mortem revealed signs of disseminated intravascular coagulation. Of the 33 hysterectomies, 14 women (43%) had only one relook laparotomy, 15 (45%) had two relook laparotomies, and 4 (12%) had three relook laparotomies, including the laparotomy for the hysterectomy. Thirty-eight per cent (*n* = 11/29) of women who had a TAH required a subsequent relook laparotomy, while 2 of the 4 women from the STAH group required relook. The six unknown files with missing information were the result of missing surgical records, despite their histopathology results being available.

**TABLE 3:** Surgical characteristics of women who had puerperal sepsis (*N* = 33).

Surgical procedure	Surgical characteristic	*n*	%
Hysterectomy type	Subtotal hysterectomy	4	12
	Total hysterectomy	29	88
Number of exploratory laparotomies	1	8	24
	2	15	45
	3	4	12
	Unknown	6	4

### Organisms identified in various sites

*Acinetobacter baumannii* was the only organism cultured in all four culture sites: intra-abdominal fluid, blood, urine and sputum (Table 4). In the HIV seropositive women, *A. baumannii, Enterococcus faecalis* and *Coagulase-negative staphylococcus* were the organisms most cultured, while in HIV seronegative women, cultures grew *A. baumannii, Escherichia coli* and *Klebsiella spp* (not shown). Of the two women who died, one, who was HIV seronegative, had *A. Baumannii* cultured in her blood, and *E. coli* was cultured in her urine. The second woman was HIV seropositive and cultures were negative for any organism.

**TABLE 4:** Organisms identified in various sites.

Organism	Sample site and frequency							
	Intra-abdominal fluid (*N* = 42)		Blood (*N* = 8)		Urine (*N* = 7)		Sputum (*N* = 2)	
	*n*	%	*n*	%	*n*	%	*n*	%
*Acinetobacter baumannii*	11	26	3	38	1	14	1	50
*Coagulase-negative staphylococcus*	-	-	2	25	-	-	-	-
*Staphylococcus haemolyticus*	-	-	1	13	1	14	-	-
*Escherichia coli*	8	19	-	-	2	29	-	-
*Enterobacter cloacae*	-	-	-	-	1	14	-	-
*Klebsiella species*	6	14	-	-	-	-	1	50
*Enterococcus faecalis*	6	14	-	-	-	-	-	-
*Enterococcus faecium*	3	7	1	13	-	-	-	-
*Staphylococcus aureus*	2	5	-	-	-	-	-	-
*Prevotella oralis*	1	2	-	-	-	-	-	-
*Proteus mirabilis*	1	2	-	-	-	-	-	-
*Staphylococcus lugdenensis*	1	2	-	-	-	-	-	-
*Staphylococcus epidermidis*	1	2	1	13	-	-	-	-
*Streptococcus anginosus*	1	2	-	-	-	-	-	-
*Candida albicans*	1	2	-	-	2	29	-	-

### Antibiogram of the cultured organisms

*Acinetobacter baumannii* (*n* = 16 sampled from four sites), the most common organism, was found to be sensitive to ceftazidime and colistin in 44% of women but resistant to tazobactam in 69%, Gentamycin in 56% and ceftazidime in 25% of women (Table 5). *E. coli* and *Prevotella oralis* were 80% and 100% sensitive to Augmentin, respectively. *Prevotella oralis* was the only organism sensitive to metronidazole (100%), while *E. coli* only responded at 10% sensitivity to Gentamycin. Only four Gram negative organisms were sensitive to Ampicillin, with *E. faecalis, Staph. ludgenensis* and *Strep. Anginosus* displaying total (100%) and *Staph. Aureus 80%* sensitivity to Ampicillin.

**TABLE 5:** Antimicrobial drug susceptibility for micro-organisms identified in blood, urine and intra-abdominal fluid cultures (*N* = 25).

Microorganism	Name of organism	Drug tested	Sensitive		Resistant	
			Number of samples†	%	Number of samples‡	%
Gram negative	*Acinetobacter baumannii* (*n* = 16)	Ampicillin	-	-	1	6
		Augmentin	-	-	1	6
		Ceftazidime	7	44	4	25
		Ceftriaxone	-	-	1	6
		Ciprofloxacin	1	6	-	-
		Clindamycin	-	-	1	6
		Cloxacillin	-	-	1	6
		Colistin	7	44	-	-
		Gentamycin	-	-	9	56
		Tazobactam	-	-	11	69
		Vancomycin	-	-	1	6
	*Escherichia coli* (*n* = 10)	Ampicillin	-	-	6	60
		Augmentin	8	80	2	20
		Ceftazidime	-	-	1	10
		Ceftriaxone	2	20	2	20
		Gentamycin	1	10	-	-
		Tazobactam	1	10	-	-
	*Klebsiella* (*n* = 7)	Ampicillin	-	-	4	57
		Augmentin	-	-	3	43
		Amikacin	2	29	-	-
		Ertapenem	4	57	-	-
		Imipenem	3	43	-	-
		Tobramycin	1	14	-	-
		Gentamycin	-	-	2	29
		Tazobactam	-	-	2	29
		Ceftriaxone	1	14	2	29
		Ceftazidime	-	-	2	29
	*Prevotella oralis* (*n* = 1)	Metronidazole	1	100	-	-
		Augmentin	1	100	-	-
		Ampicillin	-	-	1	100
	*Proteus mirabilis* (*n* = 1)	Augmentin	-	-	1	100
		Ceftazidime	1	100	-	-
		Gentamycin	-	-	1	100
	*Enterobacter cloacae* (*n* = 1)	Ampicillin	-	-	1	100
		Cotrimoxazole	1	100	-	-
		Amikacin	1	100	-	-
		Cefepime	1	100	-	-
		Nitrofurantoin	1	100	-	-
Gram-positive *n* = 19	*Enterococcus faecium* (*n* = 4)	Ampicillin	-	-	2	50
		Vancomycin	2	50	-	-
	*Enterococcus faecalis* (*n* = 7)	Ampicillin	7	100	-	-
	*Staphylococcus lugdenensis* (*n* = 1)	Ampicillin	1	100	-	-
		Cloxacillin	1	100	-	-
	*Staphylococcus aureus* (*n* = 2)	Ampicillin	1	50	-	-
		Cloxacillin	2	100	-	-
	*Coagulase-Negative Staphylococcus* (*n* = 2)	Vancomycin	2	100	-	-
	*Staphylococcus epidermidis* (*n* = 2)	Vancomycin	2	100	-	-
	*Staphylococcus haemolyticus* (*n* = 2)	Vancomycin	2	100	-	-
	*Streptococcus anginosus* (*n* = 1)	Ampicillin	1	100	-	-
Fungus (*n* = 2)	Candida albicans (*n* = 2)	Fluconazole	2	100	-	-

† , Number of samples expressed as a percentage of the organism in question and not its gram or fungal status.

‡ , Number of samples expressed as a percentage of the organism in question and not its gram or fungal status.

### Intraoperative findings of exploratory laparotomies

At the primary relook laparotomy, peritoneal pus was localised to the pelvis of four women (*n* = 4/13, 31%), while in one woman (8%) who had a second relook and one (8%) with a third relook laparotomy (8%) also had peritoneal pus localised in the pelvis (not shown). Four-quadrant sepsis was described in three (*n* = 3, 23%) women, and pus was described in the myometrium of one (*n* = 1, 8%) (Table 6). There was one woman whose uterine scar was bleeding. Serous fluid collection was found in 13 (*n* = 13/21, 62%) women at the time of the primary laparotomy and 8 (*n* = 8/21, 38%) in the second laparotomy. In the first relook laparotomy, the uterine scar was noted to be healthy in five (*n* = 5/22, 23%) women and necrotic in 11 (*n* = 11/22, 50%), and it was healthy in 1 (5%) and necrotic in 3 (14%) of the women at the second relook laparotomy. Additionally, the uterus was described as healthy in eight (*n* = 8/25, 32%), necrotic in three (*n* = 3/25, 12%) and atonic in 10 (*n* = 10/25, 40%) women in the first relook laparotomy. Notes for four women were not found. Localisation of pus in the pelvis and necrotic uterine scar was also noticed in the two women who had a third relook laparotomy. Other findings included pus in the myometrium in one woman and bleeding from the uterine scar in another (Table 6).

**TABLE 6:** Intraoperative findings of exploratory laparotomies.

Finding	*n*	Intraoperative finding	1st Laparotomy		2nd Laparotomy		*p* *
			*n*	%	*n*	%	
Peritoneal pus	13	-	-	-	-	-	0.67
	-	Localised to the pelvis	4	31	1	8	-
	-	Four-quadrant sepsis	3	23	0	0	-
	-	Other†	1	8	1	8	-
Serous collection	21	-	13	62	8	38	0.52
Uterine scar	22	-	-	-	-	-	0.99
	-	Healthy	5	23	1	5	-
	-	Necrotic	11	50	3	14	-
	-	Other‡	1	5	0	0	-
Uterus	25	-	-	-	-	-	0.26
	-	Healthy	8	32	1	4	-
	-	Necrotic	3	12	2	8	-
	-	Atonic	10	40	1	4	-

NB: Only two patients had a 3rd laparotomy (two with pus in pelvis and one with pus in myometrium); because of the small numbers, these have been excluded from the Fisher’s Exact Test

* , *p*-value from Fisher’s Exact Test.

† , Other: Pus in the myometrium.

‡ , Other: Bleeding.

### Other sources of sepsis

Respiratory tract infections were diagnosed in 4 (12%) women, bacteraemia in 3 (9%) and urosepsis in 2 (6%) women out of the entire cohort of 33 women who had hysterectomies. Two women (6%) died from complications of ongoing sepsis. The cause of death was identified as acute respiratory distress syndrome because of the ongoing sepsis in the one with STAH and a necrotic cervix diagnosed at post-mortem. The second woman who died of TAH had ongoing sepsis complicated by disseminated intravascular coagulopathy, with a deep incisional site infection. Ninety-four per cent of the women (31) in this study population survived and were subsequently discharged home.

### Comparison of intraoperative and histopathological findings

The histopathological diagnosis documented the presence of intra-abdominal pus in 17 of 32 samples (53%) sent for histology, while this was documented in 12 of 27 (44%) by the surgeon. Uterine necrosis was also described by the surgeon in 8 of 27 (30%) women compared to 23 of 32 (72%) uterine samples examined histopathologically, *p*-value 0.004.

### Histological findings of the uterus

Puerperal sepsis was reported in the majority (85%) of women histologically (Table 7). This was evident by the presence of suppurative inflammation and necrosis of varying degrees in the uterine tissue. The endometrium was described as inflamed (endometritis) in 6% and myometrial ischaemia in 3% of the 33 women. No histological evidence of sepsis was found in the report of one woman (3%), and there was no laboratory report for one of the histology samples because of a laboratory-related issue.

**TABLE 7:** Histological findings of the uterus to ascertain the presence or absence of sepsis (*N* = 33).

Histological finding	*n*	%
Inflamed endometrium	2	6
Myometrial ischaemia	1	3
Puerperal sepsis reported	28	85
No puerperal sepsis reported	1	3
Unknown†	1	3

† , Unknown: Specimen submitted but not reported because of a laboratory-related issue.

## Discussion

The outcomes of 33 women who underwent hysterectomy for puerperal sepsis at CHBAH during the study period are described. These women constituted 0.17% of the total (18 458 women) deliveries during this study period.

The background incidence of hypertension in pregnant women in Soweto (unpublished data) is 11%. The 15% incidence of hypertension in this study may highlight a higher level of hypertension among women with hysterectomy for puerperal sepsis. Whether this is because of the disease process or an increased frequency of iatrogenic interventions requires further investigation. This confirms previous findings of the contribution of hypertensive disease in pregnancy to placental insufficiency, but also pre-eclampsia. Iatrogenic delivery for growth-restricted foetuses via CS may contribute to a higher frequency of CS in this population, putting them at risk of sepsis associated with a CS.^18^

Total abdominal hysterectomy is a method of source control in the management of severe uterine sepsis. However, variations in technique, surgical experience and consistency of approach are not standardised but rather individualised.^9,19^ In our study, 29 women (88%) had a TAH, while 4 (12%) had a STAH. There is no existing literature supporting that a TAH is superior to an STAH in the management of puerperal sepsis. The only evidence regarding TAH and STAH was in post- and peripartum haemorrhage.^19^ The mortality rate in this study was 6% which may suggest that early source control and probably by TAH, has better outcomes.

It is widely known that the common organisms of puerperal sepsis are *E. coli, Group B streptococcus, Klebsiella pneumoniae* and *Staphylococcus species*.^20^ Our study found that all but one of the ESKAPE organisms, namely *Enterococcus faecium, Staphylococcus aureus, Klebsiella pneumoniae, A. baumannii* and *Enterobacter spp*., were the most common organisms cultured. *Pseudomonas aeruginosa* was not cultured from any of the women. With *A. baumannii* being the most frequently cultured, followed by *E. coli* and *Klebsiella*, the same organisms were mostly cultured in a study conducted in the Western Cape, South Africa.^19^ Given the paucity of evidence from South Africa on the types of organisms that are most often cultured, this study adds to the body of knowledge on the subject. All three, *A. baumannii, E. coli* and *Klebsiella* of these organisms were found to be resistant to Augmentin, Ampicillin and Gentamycin, which are commonly prescribed in our hospital facility to treat puerperal sepsis and chorioamnionitis. The resistance to Ampicillin is increasing, prompting a re-evaluation of antibiotic prophylaxis for premature rupture of membranes which contributes to puerperal sepsis. However, *E. coli* was the only ESKAPE organism with some sensitivity to Augmentin. The organisms commonly cultured in this study showed that our current prophylactic and treatment regimens are ineffective at preventing sepsis that required hysterectomy as source control measure. As most of these organisms are resistant to our current prophylactic and treatment regimens, this needs further investigation. These findings substantiate the need to use targeted antibiotic therapy in the management of puerperal sepsis and highlight the importance of antimicrobial stewardship as more organisms are becoming resistant to broad-spectrum antibiotics. *E. coli* and *A. baumannii* were cultured from one of the women who died. Whether the colonisation with extremely drug-resistant *A. baumannii* may have contributed to the demise of this woman because of inadequate antibiotic availability needs confirmation. The study was, however, not designed to ascertain whether these organisms contributed to the demise of this woman. Because of the increasing incidence of *A. baumannii* and the varying sensitivity patterns among these patients, alternative broad-spectrum antibiotic treatment options may be required.^20^

The source of sepsis was independent of HIV seropositivity in this study. It is, however, important to note the lower seropositivity rate in the current study than the background rate of HIV seropositivity of 28.1% in Gauteng.^21^ Additionally, antiretroviral treatment of the few seropositive cases was noted. At our centre, the protocol of postpartum prophylactic antibiotic use for 5 days in HIV seropositive women who had a CS may have led to no impact of HIV infection on the frequency of puerperal sepsis and hysterectomy. There was no difference in histopathological findings between HIV seropositive and seronegative women.

The findings of bacteraemia, respiratory tract and urinary tract infection highlight the importance of concurrent investigations to improve detection, as foci of infection may occur in more than one site.

The indications for hysterectomy in this institution are based on clinical features of the women, two or more organ dysfunction, necrotic cervix, pus draining from the cervix or if at laparotomy, the uterus appeared necrotic. A unique finding of this study was that histological and microbiological diagnoses are useful additions to the surgeon’s macroscopic diagnosis of necrosis and the appearance of pus, in comparison, evidence of which may improve antibiotic prophylaxis in sepsis. The indication for the hysterectomy was not based solely on the uterine appearance. There is a paucity of evidenced literature regarding the complementation of surgeons’ diagnoses of necrosis, which highlights the need for an appropriate scoring system to objectively identify women who require a hysterectomy.

### Study’s strengths

This study was conducted in a tertiary hospital, which serves as a referral centre for high-risk maternity and foetal conditions from surrounding centres, thus reflecting a low to middle-income South African population. The study population is reflective of the referring cluster and the management at a single tertiary institution. The benefit of a single centre eliminates the heterogeneity of protocols and, challenges of different institutions. The histological comparison to intraoperative surgical findings illustrates the efficient documentation because of a uniform approach to documentation in this institution. Another strength is the availability of laboratory microscopy, culturing of organisms and sensitivity testing to aid in the analysis of the antibiogram.

### Study’s limitations

The main limitation is the sample size because of the study period. Moreover, the retrospective nature of the study met some missing information, and the absence of data on other factors that may contribute to sepsis (e.g. surgical experience, delays in hospital presentation, surgery wait times and timing of laparotomy as a measure of source control) may be other limitations. The majority of the patient samples obtained were from the postpartum period. Other factors, such as prolonged labour, meconium-stained liquor, number of vaginal examinations and the length of rupture of membranes, which may contribute to the development of puerperal sepsis, were not considered in this study. While it is known that early source control contributes to better outcomes, this study was not designed to evaluate early source control. A prospective study with a larger sample size may address these limitations.

## Conclusion

In conclusion, timeous and appropriate hysterectomies as a measure of source control are required in uterine sepsis. Puerperal sepsis may result in multiple end organs being affected, motivating the use of multi-organ assessment models. This study creates awareness about the increasing number of organisms associated with puerperal sepsis, the rising antibiotic resistance in this population, suggesting a review of our prophylactic and treatment antibiotic regimens used in the population of South Africa.
